# Austerity and Abortion in the European Union

**DOI:** 10.1093/eurpub/ckw026

**Published:** 2016-03-23

**Authors:** Joana Madureira Lima, Aaron Reeves, Francesco Billari, Martin McKee, David Stuckler

**Affiliations:** 1 Department of Sociology, University of Oxford, Oxford, UK; 2 London School of Hygiene and Tropical Medicine, London, UK

## Abstract

Economic hardship accompanying large recessions can lead families to terminate unplanned pregnancies. To assess whether abortions have risen during the recession, we collected crude abortion data from 2000 to 2012 from Eurostat for countries that had legal abortions and complete data. Declining trends in abortion ratios between 2000 and 2009 have been reversing. Excess abortions between 2010 and 2012 totaled 10.6 abortions per 1000 pregnancies ending in abortion or birth or 6701 additional abortions (95% CI 1190–9240) with stronger effects in younger ages. Economic shocks may increase recourse to abortion. Further research should explore causal pathways and protective factors.

## Introduction

Whenever large, unexpected events such as economic recessions happen, the hardship they trigger can lead families to face difficult decisions, including what to do in the case of unplanned pregnancies.^1–3^ Families may choose to have fewer children, and some may also terminate unplanned pregnancies.

Although there have been anecdotal media reports that austerity and recession triggered a rise in abortions, especially in hard-hit countries such as Portugal^4^ and Spain,^5^ to our knowledge, there has yet to be a rigorous analysis of available abortion data across Europe.

## Methods

To assess whether abortions have risen during the recession, we collected crude abortion data from 2000 to 2012 from Eurostat 2014 edition, disaggregated by 5-year age bands. Countries with legal abortions and complete data for the whole study period included Bulgaria, Croatia, Czech Republic, Finland, Germany, Hungary, Latvia, Lithuania, Romania, Slovakia, Slovenia, Spain and the UK. Slovenia was exceptional in that it had data for the whole period except for 2011. To address that, we obtained a point estimate of the abortion ratio for that year using a linear combination of the estimators: Total number of abortions per conception (Eurostat), Total number of Abortions (WHO), Total Fertility Rate and GDP per capita in constant USD.

Data on the number of conceptions are, inevitably, unavailable. However, a standard way to measure trends in abortions is to compute the proportion of pregnancies, excluding those ending in miscarriages, that end in abortion,^6^ here termed “abortion ratio”. The abortion ratio is computed as abortions/(abortions + live births) (per 1000 pregnancies). Analyses were weighted to adjust for country population sizes.

Web Appendix 1 describes abortion legislation and data availability.

To calculate excess abortions we used a discontinuity design whereby a trend for 2000–9 was predicted and observed abortions were subtracted from the predicted values for the 2010–12 period. Calculations were then undertaken by country and age group.

## Results

First, we ask whether there is a rise in all-age abortion ratios over and above historical trends.

**Figure 1:**
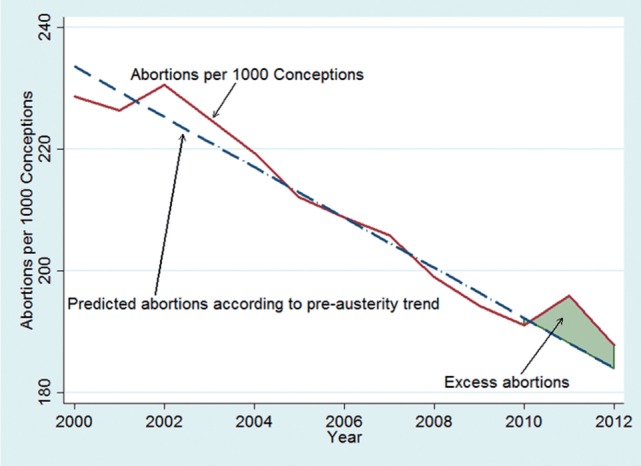
Trends in abortions rates per 1000, 13 EU countries. *Source* EUROSTAT, Population and Social Conditions Database, 2014

Figure 1 shows that abortion ratios have been declining between 2000 and 2009 across our set of European countries, but since 2009 this trend has been reversing and increasing by ∼5%. Excess abortions between 2010 and 2012 totaled 10.6 abortions per 1000 pregnancies ending in abortion or birth. In absolute numbers this means 6701 additional abortions (95% CI 1190–9240).

### Trends in abortion rates by age and country

We next disaggregated the analysis by country and maternal age groups (Figures 2 and 3 in Supplementary Web Appendix S1).

There was marked heterogeneity by country.

Excess abortions per 1000 conceptions during the austerity period were registered in Bulgaria, Czech Republic, Slovenia, Romania, Hungary, Croatia, Latvia, Slovakia and Finland albeit to very different extents. Values range from 122.4 CI (106.4–138.4) and 113.2 CI (97.2–129.2) in Bulgaria and the Czech Republic, respectively, to 20.7 CI (4.7–36.7) in Slovakia. In Finland, we observed an excess of 1.2 in the same period but this estimate was not statistically significant at the *α* = 0.05 level.

As for the remaining countries, the ratios are below what would be expected had we observed the 2000–09 trend. Estimates for Spain (−9; 95% CI −25 to 7.0) and Germany (−10.6; 95% CI −26.6 to 5.4) remained closely to their pre-austerity trend while in Lithuania −22.3 (95% CI −38.3 to −6.3) and the UK −23.6 (95% CI −39.5 to −7.6) abortion ratios declined despite austerity.

Once we focus on age groups the story becomes more complex. In most of the sample, the abortion ratios among 15–19-year-olds did not substantially deviate from the pre-austerity trend. However, in Germany and the UK, abortion ratios were lower than expected (see Supplementary WebAppendix S1) and in Bulgaria and Spain the estimates were higher than expected: the largest excesses in this age group with 108.4 and 122.9, respectively.

Among 20–24-year-olds, we observe excess abortions in Bulgaria, Latvia and Spain and fewer abortions in Lithuania and Slovenia. The 25–29-year–olds age group, however, show excess abortions in Bulgaria, Croatia, Finland, Hungary, Latvia and Slovenia.

Excess abortions among 30–34-year-olds are observed across the sample, with no exception and are statistically significant in 10 out of 13 countries. For 35–39-year-olds we also observed excess abortions for all countries—statistically significant in Croatia, the Czech Republic, Germany, Slovenia and the UK—except Lithuania where we registered a non-statistically significant decrease.

### Potential explanations

What can account for these differing trends? One possibility is that they are an artefact of the data. Declining rates of unprotected sex, intentionally or otherwise, may have led to reductions in unplanned conceptions, and thus in induced abortions. However, in the more affected countries such as Bulgaria, the total number of abortions per 1000 live births rose from 417.78 in 2010 to 447.68 in 2011 and then 433.91 in 2012.

One limitation of this initial analysis is that the data employed do not cover the entire EU. While there are gaps in data on abortion, particularly in countries where it is illegal, such as Ireland and Malta, we excluded these countries, so that the remaining sub-set had consistent data. Although differences in reporting may account for some disparities across countries, they are unlikely to account for the short-term fluctuations that we have documented. To address the possibility of a data artefact, we replicated our analysis using WHO European Health for All Database (which collects data from a more diverse set of sources than EUROSTAT, including Ministries of Health), finding consistent patterns.

Several trends are worth noting in countries which were excluded given incomplete data throughout the study period. In Portugal, abortion was legalized in 2007, and there was a substantial rise in the abortion rate thereafter. Another trend was seen in Iceland where, in the years for which data are available, there was an increase in abortions in younger women.

## Conclusions

One plausible explanation for the trend in abortions that we observed is a rise in unplanned pregnancies, which are more likely to end in abortion than planned pregnancies. Some families who, in financially stable periods, might decide against an abortion in the case of an unplanned pregnancy, might decide to terminate it when facing economic insecurity. This could also happen with planned pregnancies if there was an unanticipated economic shock, such as loss of a job in a head of household.

Another set of explanations for the observed change in the trend focuses on the role of family breakdown. It is well documented that recessions are linked to rising rates of divorce, e.g. in the South East Asia financial crisis of the late 1990s^7^ and in the current recession in the US^8^ and in the UK.^9^ Household dissolution would increase the risk of unplanned or unwanted pregnancies, by increasing the proportion of the population who are recently separated, divorced or single. Potentially compounding this is an increasing risky sexual behaviour among these groups. There is an evidence that binge drinking tends to rise in a small high-risk group during recessions,^10^ which may increase unplanned pregnancies.

These initial findings highlight the need for more detailed research on abortions and fertility declines in the context of the ongoing Great Recessions of Europe since 2007.

## Supplementary data

Key points

- The effect of the great recession on abortions in the EU has not yet been systematically scrutinized despite anecdotal evidence from the most affected countries.
- We find that declining trends in abortion rates between 2000 and 2009 have been reversing and calculate excess abortions between 2010 and 2012.
- Public policy should target the steps in the causal pathway leading a family or individual woman to decide to terminate a pregnancy that are amenable social protection, e.g. employment protection and free childcare.
